# Etablierung einer Hörscreeningzentrale zum Neugeborenen-Hörscreening im Land Baden-Württemberg

**DOI:** 10.1007/s00106-025-01717-x

**Published:** 2026-01-19

**Authors:** M. Gestewitz, M. Hornisch, P. Feyh, J. Okun, P. Beckert, I. Bruder, G. Hoffmann, P. K. Plinkert, P. J. Schuler

**Affiliations:** 1 Klinik für Hals-Nasen-Ohrenheilkunde, Universitätsklinikum Heidelberg, Im Neuenheimer Feld 400, 69120 Heidelberg, Deutschland; 2 Dietmar-Hopp-Stoffwechselzentrum, Zentrum für Kinder- und Jugendmedizin, Universitätsklinikum Heidelberg, Heidelberg, Deutschland; 3 Klinik für Allgemeine Pädiatrie, Neuropädiatrie, Stoffwechsel, Gastroenterologie, Nephrologie, Universitätsklinikum Heidelberg, Heidelberg, Deutschland; 4 Qualitätssicherung im Gesundheitswesen Baden-Württemberg (QiG BW) GmbH, Stuttgart, Deutschland

**Keywords:** Qualitätssicherung, Frühkindliche Schwerhörigkeit, Gesundheitsversorgung, Tracking, Compliance, Qualitiy assurance, Early childhood hearing loss, Health care, Tracking, Compliance

## Abstract

Seit dem 01.01.2019 erfolgt die zentrale Nachverfolgung von auffälligen Hörscreening-Ergebnissen im Land Baden-Württemberg durch eine hierzu eigens etablierte Hörscreeningzentrale für das Neugeborenen-Hörscreening mit Sitz in Heidelberg und Stuttgart. In der vorliegenden Arbeit berichten wir über die aktuelle Konzeptionierung und Umsetzung des Trackings im Bundesland. Ein Screeningprogramm ist nur dann effektiv und zielführend, wenn sämtliche Beteiligten, wie Eltern, Geburtseinrichtungen, niedergelassene Fachärzte und anderweitige Leistungserbringer, zusammenwirken. Der Arbeitsaufwand für die Aufklärung, Durchführung der Screeninguntersuchungen sowie Bestätigungsdiagnostik im vorgegeben Untersuchungszeitfenster ist immens. Ein Nachverfolgungsmechanismus im Sinne der Qualitätssicherung des Neugeborenen-Hörscreenings erscheint geboten.

## Abstract (en)

Since 01.01.2019, the central tracking of conspicuous hearing screening results in the federal state of Baden-Württemberg has been carried out by a specially established hearing screening center for newborn hearing screening based in Heidelberg and Stuttgart. In this paper, we report on the current conceptualization and implementation of tracking in the federal state. A screening program is only effective and goal oriented if all involved parties—including parents, birthing facilities, specialists in private practice, and other service providers—work together. The amount of work involved in providing information, carrying out the screening examinations, and performing confirmatory diagnostics within the specified timeframe is immense. A follow-up mechanism in terms of quality assurance for newborn hearing screening appears to be necessary.

Schätzungsweise 1 - 2 von 1000 Lebendgeborenen kommen in Deutschland mit einer Hörbeeinträchtigung mit Versorgungsnotwendigkeit zur Welt [4]. Damit zählt die frühkindliche Hörstörung zu den am häufigsten identifizierbaren Gesundheitsstörungen im Rahmen der Früherkennungsuntersuchungen bei Neugeborenen. Wird eine frühkindliche Hörstörung nicht oder zu spät erkannt, ergeben sich in Abhängigkeit vom vorliegenden Schweregrad der Hörstörung empfindliche Entwicklungseinbußen des Kindes. Die Sprachentwicklung verläuft verzögert und unvollständig, im schlimmsten Fall bleibt sie ganz aus. Das Hörempfinden stellt die Grundlage einer uneingeschränkten Kommunikationsfähigkeit des Kindes dar und ist Ausgangspunkt einer emotional, sozial und kognitiv/intellektuell altersgerechten Entwicklung [9].

Daher wurde mit Beschlussfassung des Gemeinsamen Bundesausschusses (G-BA) vom 19. Juni 2008 das Neugeborenen-Hörscreening ab dem 01.01.2009 bundesweit rechtsverbindlich in den Leistungskatalog der gesetzlichen Krankenversicherungen aufgenommen [1]. Die Installation eines entsprechenden Organs zur Kontrolle und Nachverfolgung des Neugeboren-Hörscreenings wird in der Kinderrichtlinie vom G‑BA nicht vorgesehen. Die Verantwortlichkeit zur Kontrolle und Umsetzung obliegt zunächst der Länderebene und fällt somit in den Zuständigkeitsbereich der jeweiligen Landesgesundheitspolitik.

Ein Screeningprogramm ist jedoch nur dann effektiv und zielführend, wenn sämtliche Beteiligte, d. h. Eltern, Geburtseinrichtungen, niedergelassene Fachärzte und anderweitige Leistungserbringer, stringent zusammenwirken. Eine systematische Nachverfolgung von auffälligen Untersuchungsergebnissen im Neugeborenenscreening, wie zum Beispiel der kongenitalen Hypothyreose [6] oder zystischen Fibrose [3], ist essenziell und hat nachweislich Einfluss auf eine frühzeitige Diagnose und Behandlung.

Seit dem 01.01.2019 erfolgt die zentrale Nachverfolgung von fehlenden und auffälligen Hörscreening-Ergebnissen im Land Baden-Württemberg durch eine hierzu neu etablierte Hörscreeningzentrale für das Neugeborenen-Hörscreening.

In dieser Arbeit berichten wir über die aktuelle Konzeptionierung des Trackings im Land Baden-Württemberg, die Ergebnisqualität von Screening und Nachverfolgung sowie die sich abzeichnenden Trends durch die stattgehabten Pandemiebedingungen.

## Ablauf des Neugeboren-Hörscreenings in Deutschland

Das Neugeborenen-Hörscreening in Deutschland ist in drei Stufen strukturiert:

1. Erste Stufe: In den ersten Lebenstagen des Neugeborenen (1.–3. Lebenstag) wird ein automatisches otoakustisches Emissions-Screening (AOAE, TEOAE) oder alternativ eine automatisierte Hirnstammaudiometrie (AABR) durchgeführt. Dabei werden physiologische Reaktionen der Hörverarbeitung automatisiert abgeleitet, um das Vorhandensein normaler Hörreaktionen im Innenohr zu überprüfen [8]. Bei Geburten mit berichtetem Risiko einer möglichen Hörstörung [5, 10] ist eine automatisierte Hirnstammaudiometrie (AABR) als Screening-Methode gemäß Kinder-Richtlinie obligat.
2. Zweite Stufe: Falls das Ergebnis der ersten Screeninguntersuchung auffällig oder nicht eindeutig ist, wird in der zweiten Stufe ein automatisiertes Hirnstammaudiometrie-Screening (AABR) notwendig. Hierbei werden die zentralnervösen Reaktionen auf akustische Reize automatisiert registriert, um die Funktion des Hörnervs zu überprüfen.
3. Dritte Stufe: Wenn auch das Ergebnis der zweiten Stufe auffällig ist oder Zweifel bestehen, wird bis zur 12. Lebenswoche eine weiterführende Diagnostik bei einem spezialisierten Facharzt für HNO-Heilkunde oder für Phoniatrie und Pädaudiologie durchgeführt. Diese umfasst eine ausführliche Audiometrie, eine genaue Untersuchung des Gehörgangs und des Mittelohrs, um eine genaue Diagnose zu stellen.

## Trackingprozess zum Neugeborenen-Hörscreening in Baden-Württemberg

Baden-Württemberg ist das Bundesland mit der dritthöchsten Einwohnerzahl Deutschlands. Von ungefähr 108.000 Lebendgeburten im Bundesland in 2020 [2] wurden 98 % der Neugeborenen in 81 stationären Geburtseinrichtungen (Kliniken) entbunden. Knapp 2 % der Geburten fanden „geplant außerklinisch“ statt [2].

**Abb. 1:**
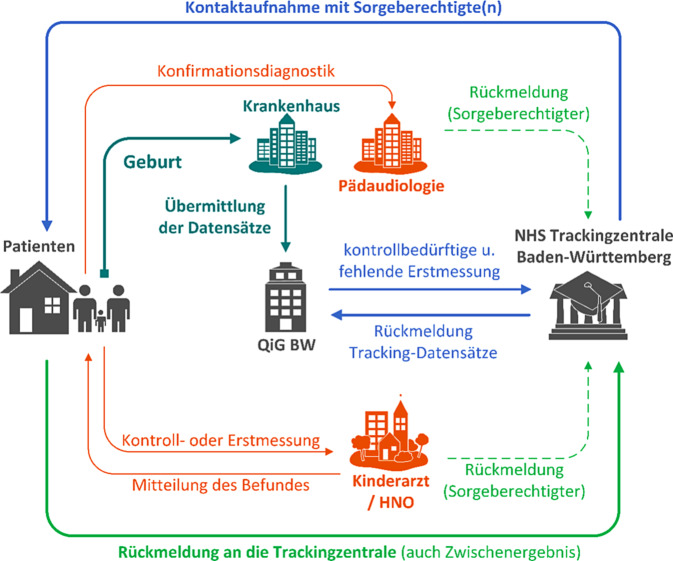
Struktur des Trackingprozesses zum Neugeborenen-Hörscreening in Baden-Württemberg. *NHS* Neugeborenen-Hörscreening

Die Hörscreeningzentrale für Baden-Württemberg setzt sich aus der Qualitätssicherung im Gesundheitswesen Baden-Württemberg GmbH (QiG BW) mit Sitz in Stuttgart und der Trackingzentrale am Dietmar-Hopp-Stoffwechselzentrum des Universitätsklinikums Heidelberg zusammen (Abb. 1).

Kommt ein Kind in einer baden-württembergischen Geburtsklinik zur Welt, wird über das Qualitätssicherungsverfahren „Universelles Neugeborenen-Hörscreening“ (QS UNHS BW) ein entsprechender Stammdatensatz zur Mutter angelegt und durch die Geburtseinrichtung mit vorliegenden Untersuchungsergebnissen des Kindes inhaltlich komplettiert. Ebenfalls ein durchgeführtes, aber auch nicht durchgeführtes Neugeborenen-Hörscreening findet in diesem Zuge seine Dokumentation.

Liegt die schriftliche Einwilligung der Sorgeberechtigten in die Datenverarbeitung vor, wird der entsprechende Datensatz zum Neugeborenen-Hörscreening an die QiG BW übermittelt. Durch die QiG BW erfolgt eine Überprüfung der eingehenden Datensätze auf Konsistenz und Vollständigkeit. Datensätze zu Kindern mit auffälligen Screening-Ergebnissen oder ohne durchgeführte Screeninguntersuchung werden an die Trackingzentrale in Heidelberg weitergeleitet.

Der aktuelle Trackingprozess (Stand: 4. Quartal 2024) gliedert sich in eine Abfolge von mehrfachen Kontaktaufnahmeversuchen zum Elternteil. Aus den von der QiG BW übermittelten Datensätzen wird zunächst ein Tracking-Datensatz generiert. Die Trackingzentrale sendet zunächst einen Briefanschreiben an die Eltern mit der Bitte die aktuell den Eltern vorliegenden Ergebnisse des Neugeborenen-Hörscreenings zu übermitteln, falls diese von der Geburtseinrichtung nicht vollständig übertragen wurden. Dies betrifft die Erstmessung, bezieht sich allerdings auch auf mögliche ambulante Folgeuntersuchungen.

Bei Ausbleiben einer Rückmeldung durch den Elternteil (z. B. telefonisch, postalisch) wird nach zwei Wochen ein erstes Erinnerungsschreiben verschickt. Bleibt die Antwort des Kontakts aus, wird nach weiteren zwei Wochen ein zweites Erinnerungsschreiben verschickt.

Nach weiteren zwei Wochen und ausbleibender Rückmeldung wird nun eine Kontaktaufnahme durch Telefonat unternommen. Gestaltet sich die Kontaktaufnahme ebenfalls frustran, wird in nach Einzelfallabwägung unter Einbeziehung bisheriger Rückmeldungen und vorliegender Testergebnisse entweder ein nochmaliges Erinnerungsschreiben (Zusatzbrief) versandt oder der Fall als „lost to follow-up“ an die QiG BW zurückgemeldet.

Im Ergebnisabschluss eines jeden Trackingfalls wird der entsprechende Tracking-Datensatz mit aktualisierten Informationen an die QiG BW zurückgemeldet. Dies ermöglicht Feedback zu den in der Summe geleisteten Neugeborenen-Hörscreenings einer jeden Geburtseinrichtung und trägt zur Qualitätssicherung bei.

## Ergebnisbetrachtung

### Geburtenabgleich und Vollständigkeit

Im Jahr 2019, der Arbeitsaufnahme der Trackingzentrale zum Neugeborenen-Hörscreening, waren der QiG BW von insgesamt 108.985 Lebendgeborenen in Baden-Württemberg [11] für 67.690 (63 %) Lebendgeborene Daten zum Universellen Neugeborenen-Hörscreening (UNHS) durch die Geburtseinrichtungen übermittelt worden. Bereits 2020 wurden der QiG BW 82.245 Datensätze (77,7 %) von insgesamt 108.024 Geburten in Baden-Württemberg gemeldet. Für das Jahr 2021 mit insgesamt 113.534 erfassten Lebendgeborenen in Baden-Württemberg wurden 88.448 Datensätze (78,9 %), im Jahr 2022 mit 104.549 Lebendgeborenen 80.988 Datensätze (79,3 %) sowie 80.666 Datensätze von 98.419 [12] Lebendgeborenen (82,7 %) zum Universellen Neugeborenen-Hörscreening im Jahr 2023 an die QiG BW gemeldet.

### Datensatzvollständigkeit

Der Anteil der auf Hörstörungen gescreenten Kinder zur Gesamtzahl der Neugeborenen soll gemäß Kinder-Richtlinie des G‑BA bei mindestens 95 % liegen. Im Jahr 2019 lag in Baden-Württemberg für 63 % der Neugeborenen ein UNHS-Datensatz vor, im Jahr 2020 für 77,7 %, im Jahr 2021 für 78,9 %. Bei aktuell vorliegendem Übermittlungsstand zeigt sich für das Jahr 2023 eine Abdeckung von 83 %. Der Anteil der auf Hörstörungen untersuchten Kinder zur Anzahl der übermittelten Datensätze lag in Baden-Württemberg im Jahr 2019 bei 94,53 %, im Jahr 2020 bei 93,67 %, im Jahr 2021 bei 93,54 % und im Jahr 2022 bei 93,85 %. Im Jahr 2023 lag der Anteil der auf Hörstörungen untersuchten Kinder zur Anzahl der übermittelten Datensätze bei 93,9 %, in Bezug zur Gesamtzahl der Neugeborenen bei 77,7 %.

Die Quote der an die Trackingzentrale übermittelten Triggerdatensätze mit fehlender Information zur Erstmessung zeigt sich konstant hoch: 2019 (48 %); 2020 (45 %); 2021 (46 %); 2022 (44,3 %); 2023 (47,4 %). Nebst Nichtdurchführung der Hörscreeninguntersuchung im Rahmen der stationären Entbindung kann dies auf eine unzureichende Datensatzpflege zurückgeführt werden. Die Diskrepanz unvollständig übermittelter Triggerdatensätze mit fehlender Angabe zur Erstmessung trotz durchgeführter Screening-Untersuchung lag exemplarisch für 2020 bei 31,2 % und für 2021 bei 24,8 %.

### Trackingdauer und Zeiten bis zur Bestätigungsdiagnostik

**Abb. 2:**
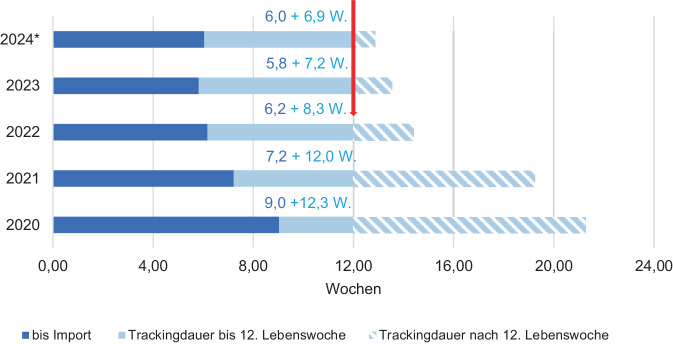
Gesamttrackingdauer von Geburt bis Fallabschluss

Die durch die Geburtseinrichtungen vorgenommene Datensatzübermittlung an die QiG BW und konsekutiv an die Trackingzentrale erfolgt aktuell im zeitlichen Mittel von 5,5 Wochen nach der Geburt des Kindes (2023). Verfahrensbedingt kann sich durch den monatlichen Übermittlungsrhythmus und den Datenfluss zwischen QiG BW und Trackingzentrale bereits eine Meldelatenz von 2–6 Wochen ergeben. Teilweise erfolgten noch vereinzelte Meldungen bis zu 20 Wochen nach dem Geburtsdatum (Abb. 2).

### Bestätigungsmethoden und Strukturqualität

Eine nach G‑BA-Richtlinie vorgesehene AABR als Erstscreening-Untersuchung bei Kindern mit berichtetem Risiko für eine Hörstörung erhielten deutschlandweit nur 40,4 % (Endbericht zur Evaluation des Neugeborenen-Hörscreenings 2011/2012).

Soweit durch das Rückmeldemodul der Geburtseinrichtungen nachvollziehbar, wurden in den Hörscreening-Verfahren bei auffälliger TEOAE 14 % mit AABR (2020), 15 % mit AABR (2021) und 17 % mit AABR (2023) kontrolluntersucht. Im Jahr 2023 wurden 75.801 Hörscreenings durchgeführt. Bei 15.392 (20,3 %) erfolgte eine AABR.

Bei den als auffällig gemeldeten Erstmessungen im Jahreszeitraum 2020/21 erfolgte eine entsprechende Kontrollmessung beim niedergelassenen Kinderarzt bzw. Facharzt für HNO-Heilkunde innerhalb von 6–7 Wochen. Bis zur erneuten Kontrollmessung vergingen durchschnittlich 11–13 Wochen, vom Erstmessungszeitpunkt ausgehend. Bei den final bestätigten beidseitigen Hörstörungen zeigte sich eine zeitliche Latenz zwischen Kontrollmessung und definitiver pädaudiologischer Zuführung und Befundverifikation im Mittel von 8–11 Wochen.

In 218 Fällen wurde zum auffälligen Hörscreeningbefund eine Kontrolluntersuchung mittels Reaktionstestung auf Geräuschstimulus (Lärmrassel etc.) vom Elternteil berichtet.

### Rückmeldung von Hörstörungen

**Abb. 3:**
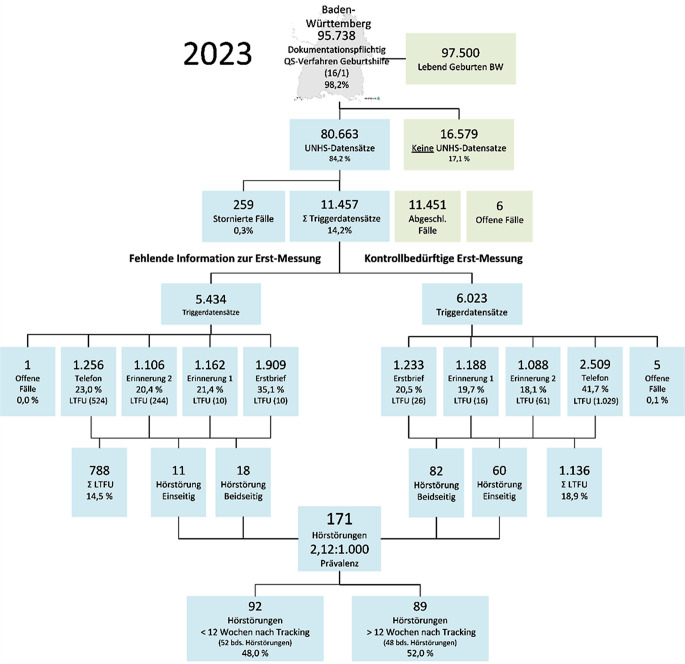
Tracking des Neugeboren-Hörscreenings Baden-Württemberg im Geburtenjahr 2023

In den Jahren 2019 bis 2022 wurde in den übermittelten Datensätzen des Neugeboren-Hörscreenings bei insgesamt 481 Neugeborenen eine Hörstörung rückgemeldet (208 einseitig; 273 beidseitig). Von Januar 2023 bis Juni 2023 wurden noch 20 Hörstörungen zu Geburten aus dem Jahr 2020 nachberichtet. Dies verdeutlicht den Zeitversatz zur Rückmeldung einer Hörstörung im Trackingprozess. Im vollständigen 5‑Jahres-Zeitraum lässt sich im Gesamtkollektiv der über das UNHS erfassten Neugeborenen in Baden-Württemberg eine Prävalenz von 1,51:1000 für berichtete Hörstörungen angeben (Abb. 3).

Eine Analyse eines großen gesetzlichen Krankenversicherers in Baden-Württemberg hat ergeben, dass die Diagnose von Schwerhörigkeit und die Versorgung mit Hörhilfen bei Kindern innerhalb der ersten 12 Lebensmonate nach der Einführung der Hörscreeningzentrale im Jahr 2019 deutlich zugenommen hat. Im Vergleich zu den fünf Jahren vor 2019 stieg die Anzahl der Diagnosen und Versorgungen signifikant an. Weitere Details zu dieser Entwicklung werden in Zukunft veröffentlicht.

### „Lost to follow up“

Die „Lost-to-follow-up-Rate“ (LTFU) beschreibt den Prozentsatz der getrackten Fälle, die im Verlauf des gesamten Trackingprozesses nicht mehr verfügbar oder erreichbar sind (z. B. Umzug) und deren endgültige Daten zum Fallabschluss daher nicht mehr erhoben werden können.

Im Jahr 2020 wurden 1448 (12,2 %) der Trackingfälle auf den Status LTFU gesetzt. In 68 % dieser Fälle konnte zumindest ein Kontakt mit der Familie hergestellt werden. In den übrigen 32 % der Fälle war es gar nicht möglich, die Familien über Brief oder Telefon zu erreichen. Ursächlich hierfür sind mutmaßlich Unzustellbarkeit der Post nach Umzug und keine oder nicht mehr aktuelle Telefonnummer im Triggerdatensatz. In 50 % der LTFU-Fälle waren mehr als drei Kontakte mit Familien zu verzeichnen. Hierunter zählten vor allem die mit der Trackingzentrale geführten Telefonate. Bei 65 % der Fälle waren drei oder teilweise mehr ausgehende Telefonate (Rückrufe, Rückfragen und Beratungsgespräche) getätigt worden. Im Jahr 2020 erfolgten insgesamt 5411 Telefonate durch die Trackingzentrale.

Der Anteil an „Lost-to-follow-up-Fällen“ am Beispiel für das Jahr 2023 beträgt bei den initial bereits als auffällig gemeldeten Datensätzen 18,7 % (1136 von insgesamt 6023 Fällen; 2023), bei den ohne vorliegende Erstmessung immerhin 14,5 % (788 von insgesamt 5434 Fällen; 2023). In Gesamtzusammenschau aller für das Jahr 2023 anfallenden UNHS-Datensätze wird eine LTFU-Rate von circa 2 % erreicht (Abb. 3).

## Diskussion

Für diese Arbeit wurde das Geburtenjahr 2023 als Betrachtungszeitraum gewählt. Dieser Zeitraum wurde ausgewählt, da die Daten für 2023 besonders zuverlässig sind. In den Vorjahren waren die Arbeitsabläufe der Hörscreeningzentrale durch die COVID-19-Pandemie und die damit verbundenen Einschränkungen, aber auch durch Prozessanpassungen nach Arbeitsaufnahme beeinflusst.

Seit Aufnahme des Trackings zum Neugeborenen-Hörscreening in Baden-Württemberg im Jahr 2019 wurden 771 Hörstörungen (Stand Q1 2024) in insgesamt 80.666 (2019–2023) Triggerdatensätzen rückgemeldet. Vor dem Hintergrund der Beobachtung von 1 bis 2 Hörstörungen auf 1000 Lebendgeborene in Deutschland [4] liegen unsere Beobachtungen im Rahmen des Screeningverfahrens in Baden-Württemberg über die Jahre zwischen 1,49 (2022) und 2,12 (2023).

Eine erste retrospektive Analyse der Daten eines großen gesetzlichen Krankenversicherers in Baden-Württemberg legt nahe, dass eine Hörscreeningzentrale für Neugeborene sinnvoll ist. Mit Einführung der Hörscreeningzentrale konnte die Anzahl der Diagnosen von Schwerhörigkeit im ersten Lebensjahr deutlich erhöht werden. Dies deutet darauf hin, dass die Hörscreeningzentrale eine wichtige Rolle bei der Früherkennung von Hörproblemen spielt.

In Hinblick auf die Datensatzqualität und Vollständigkeit bildet die Trackingzentrale einen wichtigen Monitor im Screeningprozess. Aufgezeigte Dokumentationslücken zur Erstmessung können zwei Ursachen haben: Entweder das Neugeboren-Hörscreening unterblieb in der Geburtseinrichtung. In diesem Fall ist eine Sensibilisierung der Sorgeberechtigten zur Nachholung einer entsprechenden Hörscreening-Untersuchung dringend geboten. Oder eine fehlende Erstmessungsinformation ist begründet durch lückenhafte Dokumentation oder fehlerhafte Übertragungsprotokolle der Geburtseinrichtung trotz erfolgter Untersuchung.

Bei auffälligem Untersuchungsergebnis werden eine Kontrolle und entsprechende zeitnahe Erinnerung an den Sorgeberechtigten erforderlich. Bei unauffälliger Screeninguntersuchung, aber fehlender Ergebnisübermittlung wird ebenfalls eine Trackingroutine initiiert. Der Trackingaufwand für eine unauffällige Screeninguntersuchung bindet Ressourcen und verunsichert zudem die Eltern.

Ein konstant hoher Anteil an Kontaktversuchen wird im Verlauf des Trackingprozesses auf „lost to follow up“ gesetzt, also in der Nachbeobachtung als „verloren gegangen“ gewertet. Bei frustraner postalischen Kontaktaufnahme hat sich die Option des ergänzenden Telefonats bewährt. Ein nicht unerheblicher Anteil an „Lost-to-follow-up-Fällen“ dürfte einer unzureichenden Informationsgrundlage beim Elternteil zum Hörscreening zuzuschreiben sein.

Um auf die notwendigen Schritte zu weiterführenden Untersuchungen einzugehen, bedarf es eines Optimums an Sachstandskenntnis und Eigenständigkeit der Sorgeberechtigten. Dieses vermag die Geburtseinrichtung vor dem Hintergrund einer Vielzahl anderer Untersuchungen am Neugeborenen nur eingeschränkt zu leisten.

Ein ungeklärter Status zur erforderlichen Hörscreening-Kontrolle ist heikel, da bei den bereits initial auffälligen Erstmessungen der Anteil der im Verlauf diagnostizierten Hörstörungen einen Anteil von 75 % einnimmt. Demnach sind weitere Hörstörungen in der Gruppe der auf „lost to follow up“ gesetzten Fälle zu verorten. Eine Nachbetreuung durch die Trackingzentrale stellt insofern, wenn auch nicht den Zieleinlauf, doch zumindest einen Etappensieg dar. In immerhin 68 % der Fälle konnte ein zumindest zeitweiser Kontakt zum Elternteil hergestellt werden.

„Lost to follow-up“ bedeutet nicht umsonst geleistete Arbeit. Ein erheblicher Anteil kann hinsichtlich des Neugeboren-Hörscreenings mindestens informiert, sensibilisiert und für weiterführende Abklärungsversuche zum initial auffälligen Neugeboren-Hörscreening motiviert werden.

Um eine valide Berechnung der Screeningrate des Neugeborenen-Hörscreenings zu ermöglichen, ist durch die Geburtseinrichtungen eine vollzählige Übermittlung durchgeführter Hörscreenings erforderlich. Die jährliche Diskrepanz zwischen dokumentationspflichtigem QS-Verfahren und vorliegenden UNHS-Datensätzen lässt sich mit einer unvollständigen Datensatzübermittlung der Geburtseinrichtungen erklären (Übermittlung lediglich bei auffälligem Hörscreening-Ergebnis). Ebenfalls darf angenommen werden, dass für einen Teil unauffälliger oder auffälliger Hörscreenings der Datensatzweitergabe an die Qualitätssicherung im Gesundheitswesen Baden-Württemberg (QiG BW) GmbH vonseiten der Sorgeberechtigten nicht zugestimmt wurde und sich somit der Beurteilung des Gesamtkollektivs entzieht.

Ein Abgleich der Daten des Neugeborenen-Stoffwechselscreenings mit den Daten des Neugeborenen-Hörscreenings findet in Baden-Württemberg aktuell nicht statt, könnte jedoch vor dem Hintergrund der Einführung einer allgemeinen Screening-ID für Neugeborene sinnvoll sein und Synergieeffekte schaffen. Die bisherige Korrelation der Geburtenmeldungen im Bundesland mit übermittelten UNHS-Datensätzen ist unscharf und insbesondere deutlich zeitversetzt, sodass eine Überprüfung erst jahresabschnittsweise vorgenommen werden kann.

Bei Umsetzung und Etablierung der Trackingzentrale des Neugeborenen-Hörscreenings in Baden-Württemberg zeigen sich im Bundesvergleich zu anderen Hörscreeningzentralen entsprechende Unterschiede:

Durch Vorschaltung der Qualitätssicherung im Gesundheitswesen Baden-Württemberg (QiG BW) GmbH wird eine Feedbackmöglichkeit für die screenende Geburtseinrichtung geschaffen. Auffällige Hörscreenings werden in einem anonymisierten Landesabgleich mit anderen Geburtseinrichtungen referenziert und im Individualfall zur Screeningverbesserung an das Qualitätsmanagement der jeweiligen Geburtseinrichtung rückgespiegelt.

Eine weitere Besonderheit stellt die organisatorische Anbindung der Trackingzentrale für das Neugeborenen-Hörscreening an das allgemeine Neugeborenenscreening des Landes Baden-Württemberg dar. Durch bereits etablierte Informatikstrukturen sowie ein zum Rückmeldewesen notwendiges Sekretariat ergeben sich Verbundeffekte für das Tracking.

Ein zusätzliches Charakteristikum des baden-württembergischen Trackingansatzes im Neugeborenen-Hörscreening ist der Direktkontakt eines „Tracking-Arztes“ zur Beratung von Elternteilen, aber auch zur Beratung von Hörscreening-Durchführenden.

Nach § 55 Absatz (2) haben die Leistungserbringer des Neugeborenen-Hörscreenings ab dem 1. Januar 2009 einmal im Kalenderjahr eine Sammelstatistik zu erstellen. Hier sollen unter anderem die Gesamtzahl der Neugeborenen (nur im Krankenhaus zu erfassen) sowie die Anzahl der im Rahmen des Neugeborenen-Hörscreenings getesteten Neugeborenen differenziert nach auffälliger TEOAE oder auffälliger AABR entsprechend einseitigem oder beidseitigem Vorkommen dokumentiert werden. Allerdings hat die Folgeevaluation des Neugeborenen-Hörscreenings 2017/2018 [7] gezeigt, dass die Sammelstatistik nicht genug Informationen liefert, um eine genaue Diagnose stellen zu können. Die Hörscreeningzentrale nimmt hierbei eine wichtige Rolle war. Sie steht kontinuierlich mit den Eltern in Kontakt und schlägt somit eine Brücke zwischen dem Screening und der eigentlichen Diagnose.

## Fazit für die Praxis

- Die Etablierung einer Trackingzentrale dient der Nachverfolgung von initial auffälligen sowie nicht erfolgten Neugeborenen-Hörscreening-Untersuchungen.
- Sie ist eine konsequente Ergänzung zum Minimaltrackingansatz des Vorsorgeuntersuchungshefts (gelbes U‑Heft) für das Kind, die Sorgeberechtigten sowie die Behandelnden.
- In Anbetracht des aufgezeigten hohen Nachkontrollbedarfes zur Hörscreening-Untersuchung sowie einer möglichst niedrigen „Lost-to-follow-up-Rate“ ist die Nachverfolgung zur Screeninguntersuchung nicht als Annehmlichkeit im Gesamtprozess zu verstehen, sie stellt ein Erfordernis zur Komplettierung des Neugeborenen-Hörscreenings dar.
- Tracking informiert den Elternteil über eine notwendige Kontroll- bzw. Bestätigungsuntersuchung.
- Ebenso leistet Tracking eine inhaltliche Unterstützung für die beteiligten Akteure zur Nachschärfung der Zielsetzungen eines Neugeborenen-Hörscreenings und schafft Behandlungskonformität sowie Compliance.
- Die Trackingzentrale sensibilisiert die entsprechenden Beteiligten hinsichtlich des Untersuchungsergebnisses und einer erforderlichen Kontrolle und motiviert für eine zielgerichtete Zuführung zu entsprechenden weiterführenden Instanzen der Bestätigungsdiagnostik.
- Tracking stellt eine erforderliche Kontrolluntersuchung im Individualfall des Neugeborenen-Hörscreenings sicher und bildet zum Gesamtaufkommen der Geburten und erfolgter Hörscreenings im Bundesland einen unverzichtbaren Monitor des Massenscreenings.

## Data Availability

Die in dieser Veröffentlichung erhobenen Datensätze können auf begründete Anfrage beim Korrespondenzautor angefordert werden.
